# Prevalence of Fragile X-Associated Tremor/Ataxia Syndrome in Patients with Cerebellar Ataxia in Japan

**DOI:** 10.1007/s12311-021-01323-x

**Published:** 2021-09-09

**Authors:** Yujiro Higuchi, Masahiro Ando, Akiko Yoshimura, Satoshi Hakotani, Yuki Koba, Yusuke Sakiyama, Yu Hiramatsu, Yuichi Tashiro, Yoshimitsu Maki, Akihiro Hashiguchi, Junhui Yuan, Yuji Okamoto, Eiji Matsuura, Hiroshi Takashima

**Affiliations:** 1 Department of Neurology and Geriatrics, Kagoshima University Graduate School of Medical and Dental Sciences, Kagoshima, Japan; 2 Faculty of Medicine, Kagoshima University, Kagoshima, Japan; 3 Division of Neurology, Kagoshima City Hospital, Kagoshima, Japan; 4 Department of Physical Therapy, School of Health Sciences, Faculty of Medicine, Kagoshima University, Kagoshima, Japan

**Keywords:** FXTAS, *FMR1* premutation, Cerebellar ataxia

## Abstract

The presence of fragile X mental retardation 1 (*FMR1*) premutation has been linked to patients with a certain type of cerebellar ataxia, the fragile X-associated tremor/ataxia syndrome (FXTAS). However, its prevalence in Japan has yet to be clarified. The aim of the present study is to determine the prevalence of FXTAS in Japanese patients with cerebellar ataxia and to describe their clinical characteristics. DNA samples were collected from 1328 Japanese patients with cerebellar ataxia, referred for genetic diagnosis. Among them, 995 patients with negative results for the most common spinocerebellar ataxia subtypes were screened for *FMR1* premutation. Comprehensive clinical and radiological analyses were performed for the patients harbouring *FMR1* premutation. We herein identified *FMR1* premutation from one female and two male patients, who satisfied both clinical and radiological criteria of FXTAS (0.3%; 3/995) as well. Both male patients presented with high signal intensity of corticomedullary junction on diffusion-weighted magnetic resonance imaging, a finding comparable to that of neuronal intranuclear inclusion disease. The female patient mimicked multiple system atrophy in the early stages of her disease and developed aseptic meningitis with a suspected immune-mediated mechanism after the onset of FXTAS, which made her unique. Despite the lower prevalence rate in Japan than the previous reports in other countries, the present study emphasises the necessity to consider FXTAS with undiagnosed ataxia, regardless of men or women, particularly for those cases presenting with similar clinical and radiological findings with multiple system atrophy or neuronal intranuclear inclusion disease.

## Introduction

The fragile X-associated tremor/ataxia syndrome (FXTAS) is an age-dependent neurodegenerative disorder caused by a 55–200 CGG repeat expansion (i.e. premutation) in the 5′ untranslated region of the fragile X mental retardation 1 (*FMR1*) gene, where the normal range is 5–40 CGG repeats. *FMR1* premutation also causes fragile X-associated primary ovarian dysfunction (FXPOI) and fragile X-associated neuropsychiatric disorders (FXAND). Additionally, more CGG repeat expansion of this region (> 200 repeats; i.e. full mutation) leads to fragile X syndrome, a well-known cause of inherited intellectual disability and autism spectrum disorder [1]. The core presenting features of FXTAS include intention tremors and cerebellar ataxia, typically beginning after the age of 60 years. Additional variable features include parkinsonism, cognitive impairment, neuropathy, and autonomic dysfunction. Therefore, FXTAS is often mistaken for other movement disorders, such as spinocerebellar degeneration, multiple system atrophy (MSA), Parkinson disease, and essential tremors. Magnetic resonance imaging (MRI) exhibit several characteristic findings, such as the middle cerebellar peduncle (MCP) sign, brain atrophy, and cerebral white matter lesions, which are all included in the diagnostic criteria [2]. Although FXTAS occurs in both male and female patients, the latter generally present with milder symptoms and lower penetration rates than the former, owing to a healthy X chromosome that does not carry the premutation. The prevalence of FXTAS in the general population has been estimated at 1/4000 and 1/7800 in men and women over the age of 55 years, respectively, although such rates can vary on account of race and ethnicity [3, 4]. In Japan, premutation allele in *FMR1* gene has not been detected from normal or autism populations [5–7], and only a few case reports have been available for FXTAS [8, 9], with no clear descriptions regarding prevalence. FXTAS is being gradually recognised in Europe and the USA. In Japan, however, FXTAS possibly remains under-diagnosed, given its rarity, lack of recognition, and overlap of symptoms with other neurodegenerative disorders, such as spinocerebellar ataxia, MSA, and progressive supranuclear palsy. Therefore, the present study attempts to clarify the prevalence of FXTAS in patients with cerebellar ataxia in Japan. Among 1328 patients with cerebellar ataxia, *FMR1* premutation was detected from three cases, and their clinical and radiological characteristics are described.

## Patients and Methods

We examined 1328 Japanese patients (from 1294 families) with chronic progressive cerebellar ataxia, who were referred to our department for genetic testing, from January 2000 to September 2020. Blood samples were collected from medical clinics/institutions in western Japan, primarily from the Kyushu region (Kagoshima, Miyazaki, Oita, Fukuoka, and Okinawa Prefectures) and Ehime Prefecture. Genomic DNA was extracted using QIAGEN’s Puregene Core Kit C (QIAGEN, Valencia, CA) according to the manufacturer’s protocol. Initially, samples were screened for repeat expansions associated with spinocerebellar ataxias (SCA1, SCA2, SCA3, SCA6, SCA7, SCA8, SCA12, SCA17, SCA31, and DRPLA) and a Pro102Leu mutation in *PRNP* gene responsible for Gerstmann–Sträussler–Scheinker syndrome (GSS-P102L) following a previously described protocol [10]. Subsequently, 333 (25.1%) patients who were found positive for repeat expansion in SCA genes or GSS-P102L were excluded, whereas the remaining 995 patients were screened for *FMR1* premutation. Screening for the CGG repeat expansion of the *FMR1* gene was performed by a two-step polymerase chain reaction (PCR) protocol with a specific primer pair (forward primer, 5′- GCTCAGCTCCGTTTCGGTTTCACTTCCGGT-3′, and reverse primer, 5′- AGCCCCGCACTTCCACCACCAGCTCCTCCA-3′) [11]. The PCR cycling profile was as follows: denaturation at 98 °C for 10 min, 10 cycles at 97 °C for 35 s, 65 °C for 35 s, and 68 °C for 4 min; 20 cycles at 97 °C for 35 s, 64 °C for 35 s, 68 °C for 4 min plus a 20-s increment for each cycle; and a final extension at 68 °C for 10 min. PCR products containing the CGG repeats were visualised using agarose gel electrophoresis followed by GelRed (Biotium Inc., USA) staining. The approximate allele size and repeat numbers were estimated via comparison with DNA ladder (BioLabs) ranging from 100 to 1517 bp. The accurate repeat numbers were determined through the Sanger method and/or fragment analysis using Peak Scanner software version 1.0 on an ABI PRISM 3130xl Genetic Analyzer (Applied Biosystems).

All patients and family members provided written informed consent to participate in this study. The study conforms with the World Medical Association Declaration of Helsinki published on the website of the Journal of American Medical Association. The study protocol was reviewed and approved by the Institutional Review Board of Kagoshima University, Japan.

## Results

The clinical characteristics of 1328 patients included in this study are summarised in Table 1. The mean age at examination and disease onset was 58.2 (standard deviation (SD) 15.3) years and 51.2 (SD 18.2) years, respectively. A total of 787 (59.3%) patients were sporadic with no family history of ataxia. All patients had cerebellar signs, 498 (37.5%) showed pure cerebellar ataxia phenotype and 580 (59.3%) showed the ‘cerebellar plus phenotype’, which is characterised by cerebellar ataxia accompanied with additional neurological features, including pyramidal signs, extrapyramidal signs, cognitive impairment, autonomic dysfunction, peripheral neuropathy, epilepsy, deafness, or psychiatric symptoms. Moreover, 116 (9.4%) patients had a clinical diagnosis of MSA before genetic testing.

**Table 1:** Clinical characteristics of patients included in the study

			**Positive patients**^a^	**Negative patients**^b^	**Total**
			***N* = 333**	***N* = 995**	***N* = 1328**
**Age at examination (years)**	Mean [SD]		57.9 [15.5]	58.3 [15.3]	58.2 [15.3]
	0–19		4 (1.2%)	22 (2.2%)	26 (1.6%)
	20–39		41 (12.3%)	82 (8.2%)	123 (9.3%)
	40–59		101 (30.3%)	339 (34.1%)	440 (33.1%)
	60–79		160 (48.0%)	488 (49.0%)	648 (48.8%)
	80-		15 (4.5%)	37 (3.7%)	52 (3.9%)
	Unknown		12 (3.6%)	27 (2.7%)	39 (2.9%)
**Sex**	Male		170 (51.1%)	540 (54.3%)	710 (53.5%)
	Female		163 (48.9%)	455 (47.7%)	618 (46.5%)
**Family history**	Positive		237 (71.2%)	223 (22.4%)	460 (34.6%)
	Negative (sporadic)		83 (24.9%)	704 (70.6%)	787 (59.3%)
	Unknown		13 (3.9%)	68 (6.8%)	81 (6.1%)
**Clinical features**	Pure cerebellar ataxia		168 (50.5%)	330 (33.2%)	498 (37.5%)
	Cerebellar plus phenotype		81 (24.3%)	499 (50.2%)	580 (43.7%)
		Pyramidal signs	30	157	187
		Extrapyramidal signs	12	164	176
		Cognitive impairment	26	173	199
		Autonomic failure	4	117	121
		Neuropathy	8	46	54
		Epilepsy	13	24	37
		Deafness	1	10	11
		Psychiatric symptoms	8	35	43
	Unkown		84 (25.2%)	166 (16.6%)	250 (18.8%)

^a^Patients who were positive for SCA 1, 2, 3, 6, 7, 8, 12, and 31, DRPLA, or GSS-P102L

^b^Patients who were negative for SCA 1, 2, 3, 6, 7, 8, 12, and 31, DRPLA, and GSS-P102L

*SD*, standard deviation

**Fig. 1:**
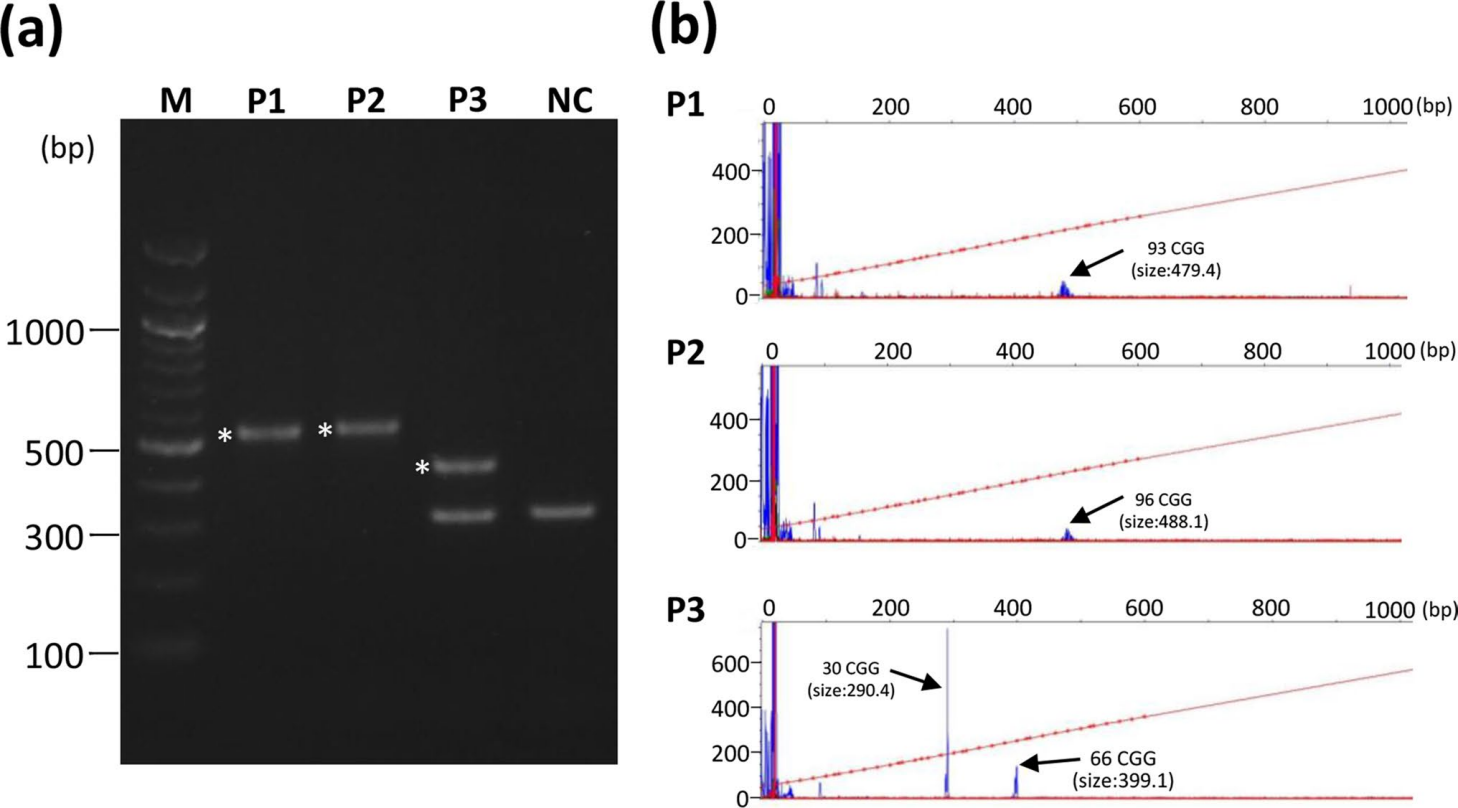
Agarose gel electrophoresis and electropherograms of polymerase chain reaction products. **a** The results of agarose gel electrophoresis of polymerase chain reaction fragments obtained along the CGG repeated region of normal male control (NC), patient 1 (P1), P2, and P3. P1 and P2 lanes showed a band around 550 bp, which corresponds to the premutation allele size (asterisks). P3 lanes showed two bands, normal allele size, and premutation allele size (asterisks). **b** Electropherograms showing the sizes of the CGG repeat alleles in each patient. The peaks representing the *FMR1* CGG repeat fragments are indicated by arrows. *X*-axis, fragment sizes in base pairs; *Y*-axis, relative fluorescence units; red line across the electropherograms, slope threshold for peak start/end. NC, normal control; *M*, maker (DNA ladder)

After excluding 333 patients with known pathogenic mutations in the SCA genes or GSS-P102L, 995 patients remained, among whom three unrelated patients (two males and one female) were identified to have the *FMR1* premutation. The PCR amplification results of the 5′-untranslated region of *FMR1* gene are shown in Fig. 1a. Fragment analysis revealed that the number of expanded CGG repeats in these three patients was 93, 96, and 66, respectively (Fig. 1b). Clinical, genetic, and radiological findings of them are summarised in Table 2.

**Table 2:** Clinical and neuroradiological findings of patients with *FMR1* premutation

		Patient 1	Patient 2	Patient 3
Sex		Male	Male	Female
Age at examination (year)		73	83	68
Age at onset (year)		63	74	63
No. of CGG triplets		93	96	30, 66
Clinical features				
	Gait ataxia	+	+	+
	Dysarthria	+	+	+
	Dysmetria	+	+	+
	Dysphagia	−	+	+
	Intention tremor	+	+	+
	Parkinsonism	+	+	+
	Tendon reflexes	n/a	Decreased	Increased
	Neuropathy	n/a	+	−
	Urinary incontinence	+	+	+
	Orthostatic hypotension	−	−	+
	Cognitive decline	+	+	+
Neuroradiological findings				
	Lesions in MCPs	+	+	±
	Lesions in the splenium of the corpus callosum	+	+	−
	Cerebral white matter lesions	+	+	−
	Cerebral atrophy	+	+	+
	Cerebellar atrophy	+	+	+
	High-intensity of corticomedullary junction on DWI	+	+	−
Systemic diseases		None	Hypertension, diabetes mellitus, hyperlipidaemia, cataract, glaucoma	Aseptic meningitis, pancreatic cysts, renal cysts

*n/a*, not available

### Patient 1

**Fig. 2:**
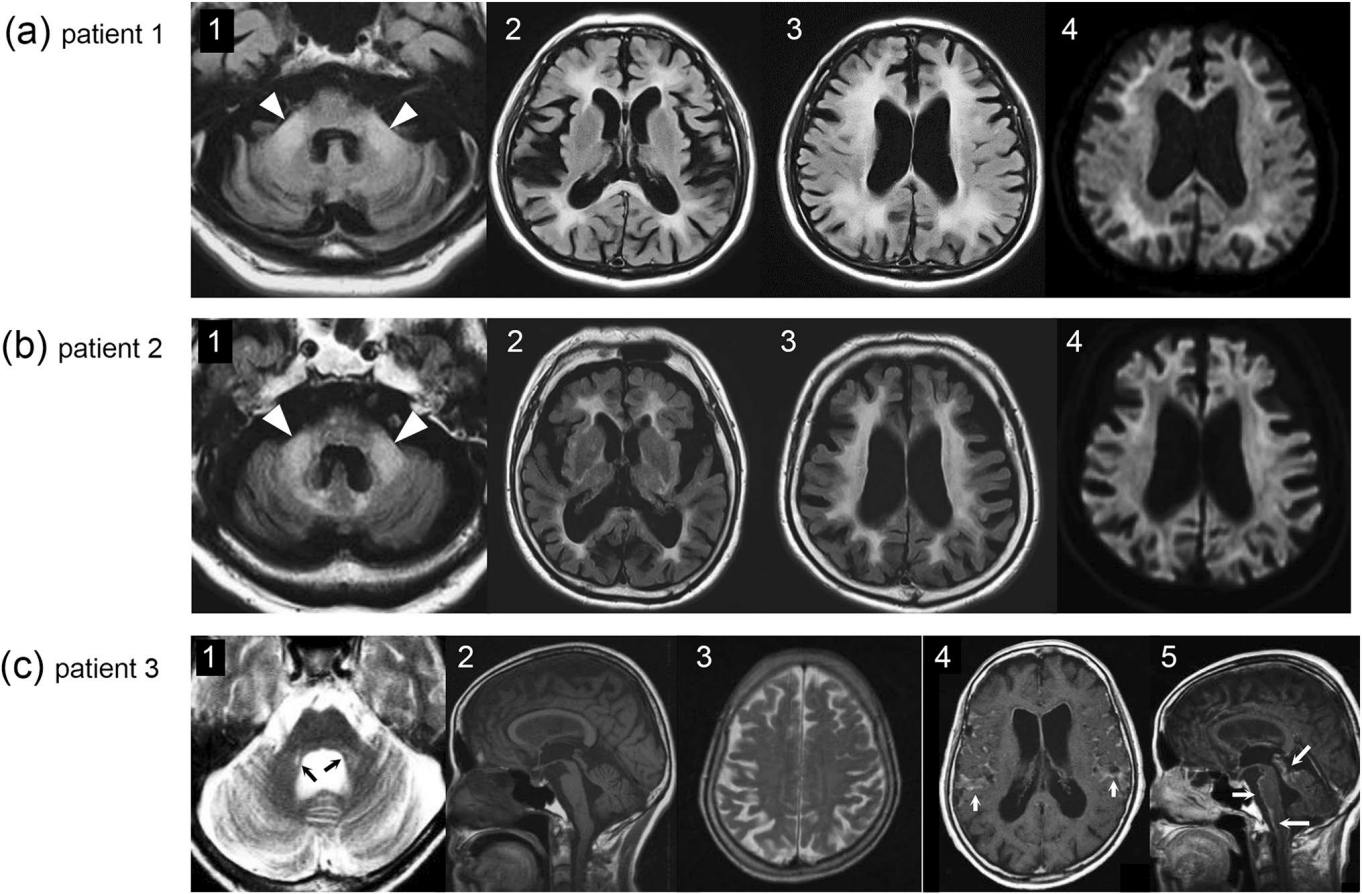
Magnetic resonance imaging (MRI) findings. Brain MRI of (**a**, **1**–**4**) patient 1 at the age of 73 years, (**b**, **1**–**4**) patient 2 at the age of 83 years, (**c**, **1** and **2**) patient 3 at the age of 68 years, and (**c**, **3** and **4**) patient 3 at the age of 69 years. Axial fluid-attenuated inversion recovery images of patients 1 and 2 showing high-intensity lesions in the (**a**, **1**; **b**, **1**) bilateral middle cerebellar peduncles (‘MCP sign’) as indicated by white arrowheads, **(a**, **2**; **a**, **3**) corpus callosum and (**a**, **3**; **b**, **3**) cerebral white matter. Moreover, a linear high signal intensity in the corticomedullary junction on diffusion-weighted imaging can be observed (**a**, **4**; **b**, **4**). Axial and sagittal images from patient 3 show (**c**, **1**–**3**) atrophy of the pons, cerebellum, and cerebral cortex with a (**c**, **1**) faint MCP sign (black arrows). At the age of 69, brain gadolinium-enhanced MRI showed (**c**, **4**; **c**, **5**) leptomeningeal enhancement of the meninges around the brainstem and cerebellum (white arrows)

A 73-year-old male patient without any past medical and family history of interest exhibited difficulty of handwriting due to tremors when he was 63 years old. At the age of 68, he was diagnosed with Parkinson’s disease by the local physician and was treated with L-DOPA, but no clinical effects were observed. At the age of 73 years, he visited a neurologist due to slowly progressive tremors, memory disturbances, and unsteady gait. Neurological examination revealed dysarthria, intention tremors, dementia, truncal ataxia, and dysmetria in both the upper and lower extremities. The Hasegawa Dementia Scale-Revised (HDS-R; 0–30 scale, normal > 20) score was 6 which indicated severe cognitive impairment. Brain MRI showed high signal intensities in the middle cerebellar peduncle (MCP), which is called as the MCP sign, and deep white matter on fluid-attenuated inversion recovery imaging with linear high signal intensity in the corticomedullary junction on diffusion-weighted imaging (DWI) (Fig. 2a). Dementia and cerebellar ataxia progressed gradually, and he was unable to walk and feed himself by the age of 74 years. He died of infectious disease at the age of 76 years.

### Patient 2

An 83-year-old male patient, who was originally noticed of having slight gait unsteadiness and cognitive impairment at the age of 74 years, was referred to the neurological department. His neurological features included dementia, dysarthria, hearing loss, action tremor, cerebellar ataxia, parkinsonism, peripheral neuropathy, and autonomic dysfunction. The aforementioned symptoms progressively worsen to the point that he had difficulty in walking and arising from a seated position without assistance at the age of 83 years, and his HDS-R score was 6. Nerve conduction studies showed mixed axonal demyelinating sensorimotor neuropathy; mild to moderate decrease in compound muscle action potential; and sensory nerve action potential in the lower limb nerves with slowing of conduction velocity and prolonged F-wave latency in lower limb nerves. On brain MRI, fluid-attenuated inversion recovery images showed the MCP sign and cerebral white matter hyper-intensity with mild linear high signal intensity in the corticomedullary junction on DWI (Fig. 2b). His daughter had a history of premature menopause and infertility, suggesting FXPOI, although screening for *FMR1* premutation was not available.

### Patient 3

A 68-year-old female patient with no family history of fragile X disorders started to show slowly progressive dizziness, gait unsteadiness, dysarthria, and hand tremors since she was 63. She was referred to our hospital at the age of 68 years, with neurological examination showing slurred speech, limb and truncal ataxia, intention tremors, rigidity, pyramidal tract sign, and cognitive impairment. Her Mini-Mental State Examination (MMSE) (0–30 scale, normal > 24) score was 17. Brain MRI showed atrophy of the pons, cerebellum, cerebral cortex, and middle cerebellar peduncle with a faint MCP sign (Fig. 2c). She was clinically diagnosed with MSA, given the predominant cerebellar ataxia and fulfilling the criteria for probable MSA according to the consensus diagnostic criteria [12]. At the age of 69 years, she developed a urinary tract infection followed by aseptic meningitis of unknown origin. On examination, the patient had no fever, headache, or neck stiffness. Brain and spinal gadolinium-enhanced MRI showed leptomeningeal enhancement of the meninges around the brainstem, cerebellum, and spinal cord (Fig. 2c). Laboratory tests revealed normal white blood cell count, C-reactive protein, and angiotensin-converting enzyme and negative blood culture and antibodies for autoimmune diseases. Cerebrospinal fluid (CSF) analysis revealed mild lymphocytic pleocytosis (15 cells/mm^3^) with increased protein (320 mg/dL), normal glucose concentration (104 mg/dL), and negative CSF cytology. Herpes simplex virus DNA and bacterial and fungal cultures of the CSF were negative. None of the findings of whole-body CT, ultrasonography, gastrointestinal endoscopy, and fluorodeoxyglucose positron emission tomography suggested malignancy. As such, a diagnosis of aseptic meningitis of unknown origin was considered, for which she received methylprednisolone pulse therapy followed by oral prednisolone (30 mg/day), which promoted rapid improvement in pleocytosis on CSF analysis and leptomeningeal lesions on MRI. However, the patient’s neurological symptoms gradually worsened. Her father and sister became bedridden in their sixties. Unfortunately, patient 3 did not attend follow-up appointments after the genetic testing; thus, we were unable to perform neurological examination or *FMR1* premutation screening on her family members.

## Discussion

The present study focused on the prevalence and characteristic clinical features of FXTAS in Japanese patients with cerebellar ataxia. Two male patients exhibited typical clinical and neuroradiological characteristics of FXTAS, which included tremors and ataxia with characteristic MRI findings (e.g. the MCP sign, brain atrophy, and cerebral white matter lesions). These findings were consistent with the definitive diagnostic criteria [2]. Notably, the brain MRI of patients 1 and 2 showed a high-intensity signal along the corticomedullary junction on DWI, which has been described as a characteristic MRI finding of neuronal intranuclear inclusion disease (NIID). Studies have speculated the presence of a significant overlap in the pathogenesis of NIID and FXTAS given their similar symptoms [13], neuroimaging findings (e.g. the MCP sign and high-intensity signal along the corticomedullary junction on DWI) [14, 15], molecular genetic ethology (expanded CGG repeats) [13, 16], and histopathology (eosinophilic intranuclear inclusions) [13, 17]. Therefore, given that FXTAS can mimic NIID (and vice versa), we highlight the need for considering FXTAS as a differential diagnosis of NIID. Our three patients were confirmed to be negative for the CGG repeat extension in *NOTCH2NLC* using the repeat-primed PCR method described previously [18]. On the other hand, in addition to the 3 FXTAS patients, we identified 8 patients with MCP sign in our cohort, comprising one with SCA2, three with clinical diagnosis of MSA-C, and four with undiagnosed SCA. Although the MCP sign indicates the diagnosis of FXTAS or can help in narrowing the differential diagnosis, it should be noted that this sign is not a disease-specific finding, as it may also be observed in certain neurodegenerative, metabolic, demyelinating, or inflammatory diseases [19].

Our female patient showed two major clinical features (intention tremors and cerebellar ataxia) and one major radiological finding (MCP sign), which fulfilled the definitive diagnostic criteria [2]. However, she had been clinically diagnosed with MSA rather than FXTAS considering that she was female and the difficulty in recognising her MCP sign. FXTAS is an age-dependent neurodegenerative disorder most commonly affecting males with the premutation, with its prevalence increasing with age from 17% in those at their 50 s to 75% in those at their 80 s [20]. In contrast, estimates have shown that approximately 12–16% of female carriers have suffered from FXTAS [21, 22]. Interestingly, a recent study indicated that female premutation carriers with FXTAS exhibit a milder phenotype than their male counterparts [23]. Additionally, previous MRI studies have indicated that female premutation carriers with FXTAS had lesser pronounced reductions in cerebellar volume and a lower incidence of the MCP sign than their male counterparts [23, 24]. Similar to previous reports, our female patient had a faint MCP sign and few cerebral white matter lesions. Female FXTAS can be assumed to have been under-diagnosed given that it often presents with atypical clinical radiological features. Therefore, more female patients would help enlighten neurologists on the importance of not overlooking female FXTAS. Surprisingly, our female patient developed aseptic meningitis during the course of her disease, and her rapid response to steroid therapy suggested an immune-mediated mechanism. Previous studies have indicated that females who had *FMR1* premutation with or without FXTAS exhibited higher incidences of immune-mediated disorders, such as autoimmune thyroid disease, fibromyalgia, irritable bowel syndrome, rheumatoid arthritis, systemic lupus erythematous, and multiple sclerosis, than age-matched controls [21, 25–28]. Although the reason why females carriers have an increased propensity for immune-mediated disorders remains unclear, various mechanisms have been suggested, such as dysregulation of specific miRNA due to sequestration of DROSHA and DGCR8 [29] or upregulation of heat shock proteins (e.g. Hsp70 and αβ-crystallin) [30], which themselves may stimulate immune dysregulation [31]. Despite to our best knowledge, this is the original report of FXTAS accompanied with aseptic meningitis; we suspect that similar immune-mediated mechanisms have been involved in the development of central nervous systems in this case.

Individuals with *FMR1* premutation may develop various symptoms related to fragile X-related diseases. Male patients with FXTAS transmit their *FMR1* premutation expansion to all of their daughters, who will be heterozygous carriers, leading to an increasing risk of having FXTAS, FXPOI, and FXAND. Moreover, premutation from female carriers can expand to full mutation in their offsprings, ultimately giving rise to fragile X syndrome. The daughter of patient 2 had a history of premature menopause and infertility, suggesting the diagnosis of FXPOI. Unfortunately, she had missed the opportunity to receive appropriate treatment like hormone replacement and an accurate genetic counselling during her gestational age. In families with *FMR1* premutation or full mutation, detailed family history review is essential for not only early diagnosis and better understanding of Fragile X-related diseases, but also for effective treatment and more precise genetic counseling.

The previously reported prevalence rates of FXTAS in populations with movement disorder are quite variable, ranging from 0 to 5% (Table 3) [32–50]. A meta-analysis revealed that approximately 1 in 300 females and 1 in 850 males in the general population had the *FMR1* premutation, whereas race/ethnic differences have also been noted [51]. In particular, three different studies in Japan found no premutation allele in normal or autism populations [5–7], suggesting a lower prevalence of the premutation allele in Japan. In fact, the present study showed a FXTAS prevalence of 0.30% (3/995) among those with ataxia after removing the patients who were negative for the most common SCA subtypes and GSS-P102L.

**Table 3:** *FMR1* gene premutation frequency in patients with cerebellar ataxia

References	Origin	Sample features	Sex	Premutation rate (%)
MacPherson et al., 2003 [32]	British	Negatives for SCA 1, 2, 3, 6, and 7	M	2/59 (3.39%)
Tan et al., 2004 [33]	Asia	Negatives for SCA 1, 2, 3, 6, 7, 8, and 10, DRPLA, and FRDA	M, F	0/55 (0%)
Milunsky et al., 2004 [34]	America	Negatives for SCA 1, 2, 3, 6, 7, 12, and 17 and DRPLA	M	1/167 (0.60%)
Zuhlke et al., 2004 [35]	Germany	Negatives for SCA 1, 2, 3, 6, 7, 12, and 17	M, F	1/510 (0.20)
Yabe et al., 2004 [36]	Japan	MSA-C	M, F	0/58 (0%)
Van Esch et al., 2005 [37]	Flanders	Negatives for SCA 1, 2, 3, 6, and 7	M	5/122 (4.10%)
Kerber et al., 2005 [38]	America	Late onset cerebellar ataxia	M, F	0/38 (0%)
Seixas et al., 2005 [39]	America	Negatives for SCA 1, 2, 3, 6, 7, 8, and 12, HD, HDL2, and DRPLA	M, F	1/233 (0.43%)
Brussino et al., 2005 [40]	Italia	Negatives for SCA 1 and 2 and FRDA	M	6/275 (2.18%)
Kraft et al., 2005 [41]	Canada	Adult onset SCA	M, F	0/69 (0%)
Biancalana et al., 2005 [42]	France	MSA or related phenotypes Negatives for SCA 1, 2, 3, 6, and 7, FDRA, and DRPLA	M, F	2/123 (1.63%)
Rodriguez Revenga et al., 2007 [43]	Spain	Negatives for SCA 1, 2, 3, 6, 7, and 8 and DRPLA	M, F	3/154 (1.95%)
Rajkiewicz et al., 2008 [44]	Poland	Negatives for SCA 1, 2, 3, 6, 7, 8, 12, and 17 and DRPLA	M	1/269 (0.37%)
Adams et al., 2008 [45]	America	Negatives for SCA 1, 2, 3, 6, and 7 and DRPLA	M	1/286 (0.35%)
Reis et al., 2008 [46]	Brazil	Ataxia, tremor, or parkinsonism	M	0/66 (0%)
Wardle et al., 2009 [47]	British	Chronic progressive cerebellar ataxia	M, F	1/178 (0.56%)
Aydin et al., 2018 [48]	Germany	Negatives for SCA 1, 2, 3, 6, 7, and 17	M, F	1/440 (0.23%)
Park et al., 2019 [49]	Korea	Tremor with cerebellar signs or extrapyramidal signs	M, F	2/74 (2.70%)
Pešić et al., 2021 [50]	Serbia	Negatives for SCA 1, 2, 3, 6, 7, and 17 and FDRA	M, F	2/100 (2.0%)
**Present study**	**Japan**	**Negatives for SCA 1, 2, 3, 6, 7, 8, 12, 31, DRPLA and GSS**	**M, F**	**3/995 (0.30%)**

*SCA*, spinocerebellar ataxia; *DRPLA*, dentatorubural pallidoluysian atrophy; *FDRA*, Friedreich’s ataxia; *MSA-C*, multiple system atrophy with predominant cerebellar ataxia; *DH*, Huntington’s disease; *DHL2*, Huntington’s disease-like 2; *GSS*, Gerstmann–Sträussler–Scheinker syndrome; *M*, male; *F*, female

The above findings suggest that the prevalence of FXTAS and allele distribution in Japanese population is lower than that in other populations. All three patients herein identified with FXTAS were residents of the Kagoshima Prefecture, Japan, which has a population of approximately 1.6 million (January 2020). Therefore, the cumulative prevalence of FXTAS in the Kagoshima Prefecture can be estimated at 0.2 per 100,000 individuals. However, the actual prevalence of FXTAS might be higher given that no screening for *FMR1* premutation was conducted in patients with other movement disorders, such as those with essential tremors or parkinsonism.

## Conclusion

On the basis of a large cohort of patients clinically presented with chronic progressive cerebellar ataxia, we identified three patients with *FMR1* premutation. Meanwhile, the prevalence of FXTAS in our cohort, 0.3% (3/995), was found lower than that of previously reports from other countries. The atypical clinical phenotypes of FXTAS, particularly in the female patients, highlighted the necessity of genetic testing of *FMR1* premutation from patients with undiagnosed ataxia.
